# Glucose Metabolism in the Progression of Prostate Cancer

**DOI:** 10.3389/fphys.2017.00097

**Published:** 2017-02-21

**Authors:** Francesca Cutruzzolà, Giorgio Giardina, Marina Marani, Alberto Macone, Alessandro Paiardini, Serena Rinaldo, Alessio Paone

**Affiliations:** ^1^Department of Biochemical Sciences “A. Rossi Fanelli”, Sapienza University of RomeRome, Italy; ^2^Department of Biology and Biotechnology “Charles Darwin”, Sapienza Università di RomaRome, Italy

**Keywords:** prostate cancer, metabolism, microenvironment, Warburg effect, inflammation

## Abstract

Prostate cancer is one of the most common types of cancer in western country males but the mechanisms involved in the transformation processes have not been clearly elucidated. Alteration in cellular metabolism in cancer cells is recognized as a hallmark of malignant transformation, although it is becoming clear that the biological features of metabolic reprogramming not only differ in different cancers, but also among different cells in a type of cancer. Normal prostate epithelial cells have a peculiar and very inefficient energy metabolism as they use glucose to synthesize citrate that is secreted as part of the seminal liquid. During the transformation process, prostate cancer cells modify their energy metabolism from inefficient to highly efficient, often taking advantage of the interaction with other cell types in the tumor microenvironment that are corrupted to produce and secrete metabolic intermediates used by cancer cells in catabolic and anabolic processes. We recapitulate the metabolic transformations occurring in the prostate from the normal cell to the metastasis, highlighting the role of the microenvironment and summarizing what is known on the molecular mechanisms involved in the process.

Alterations in cancer cell metabolism have been considered for years a secondary effect in the complex picture of tumor biology in spite of the unequivocal evidence that changes in cell function always require changes in cell metabolism. Recently, important studies focused the attention on the cellular pathways responsible for energy production and synthesis of molecules, both required to sustain the replication of cells that are growing uncontrolled. As a consequence, new promising therapeutic targets are emerging.

The proliferation of normal cells is controlled by the presence of growth factors in the microenvironment and by the interaction with other cells; oxygen and nutrients are provided by the blood. Transformed cancer cells acquire the ability to proliferate in absence of growth factors and to ignore the antiproliferative stimuli like for example those mediated by cell-to-cell contacts. Cancer cell proliferation, *per se*, moves the cells away from the blood vessels and therefore from oxygen and nutrients generating a hypoxic and nutrient-poor microenvironment. The adaptation of cancer cells to the novel situation implies a profound reprogramming of metabolism, largely affecting pathways involving the key metabolic substrates (such as glucose, but not only) and redox homeostasis. Among cell's organelles, mitochondria witness large functional changes, which reflect the novel needs of the cancer cells. Paradoxically several cancer cell types show a very high glycolytic rate, and pyruvate is not used in these cells for the Krebs cycle but transformed into lactate as a waste product in the so-called Warburg effect (Warburg, 1956), generating a very small amount of energy with respect to the complete respiratory process potential. Lactate fermentation is often used in normal cells under hypoxic conditions, but cancer cells use this metabolic pathway independently from oxygen availability. The reason for this metabolic switch in cancer cells is not entirely clear, in particular considering its low energetic efficiency, and it has been hypothesized that this pathway is used to produce metabolic intermediates needed in anabolic processes fundamental for cancer cells.

Prostate cancer is the second most common type of cancer in western country males (Siegel et al., 2016). Fortunately, 80% of patients with an early diagnosis have a very good prognosis and radical prostatectomy is still the most diffused approach with a high rate of success and no side effects. However, the prognosis becomes worse if the diagnosis is tardive and the disease becomes metastatic. Cancer lesions can develop in two different regions of the prostate gland, most commonly (80%) in the periphery zone; the remaining lesions are found in the transition zone, which is located in the periurethral region (McNeal, 1988).

The prostate has a peculiar and unique metabolism that changes during tumor onset and progression from the prostate intraepithelial neoplasia (PIN) to metastasis (see Table 1). This is in agreement with the current opinion on the heterogeneity of metabolism remodeling in cancer cells (Strickaert et al., 2016). This scenario is further complicated by the interaction between prostate cancer cells and other cell types present in the microenvironment, such as immune cells, fibroblast or adipocytes in the metastasis, actively participating in tumor metabolism. In this review we will recapitulate the metabolic transformations of glucose metabolism that occur from normal prostate epithelium to the metastatic microenvironment focusing on the molecular events that are or could be responsible for these alterations.

**Table 1:** **Metabolic alterations in prostate, breast and liver cancer cells**.

	**Prostate**			**Breast**		**Liver**
	**Normal cells**	**Cancer cells**	**Metastatic cells**	**Triple negative**	**Estrogen positive**	**Cancer cells**
Ox Phos	Inactive	Active	Inactive	Inactive	Active	Inactive
Krebs cycle	Inactive	Active	Inactive	Inactive	Active	Inactive
*Glucose*	Consumed	Consumed	Consumed	Consumed	–	Consumed
*Lactate*	–	Consumed	Accumulat.	Secreted	Imported	Secreted
*Citrate*	Secreted	Consumed	–	–	Consumed	–

*The main metabolic alterations of glucose metabolism in prostate, breast (TN and estrogen positive) and liver cancer cells are summarized (Elia et al., 2016)*.

## Citrate metabolism in prostate epithelial cells

**Figure 1:**
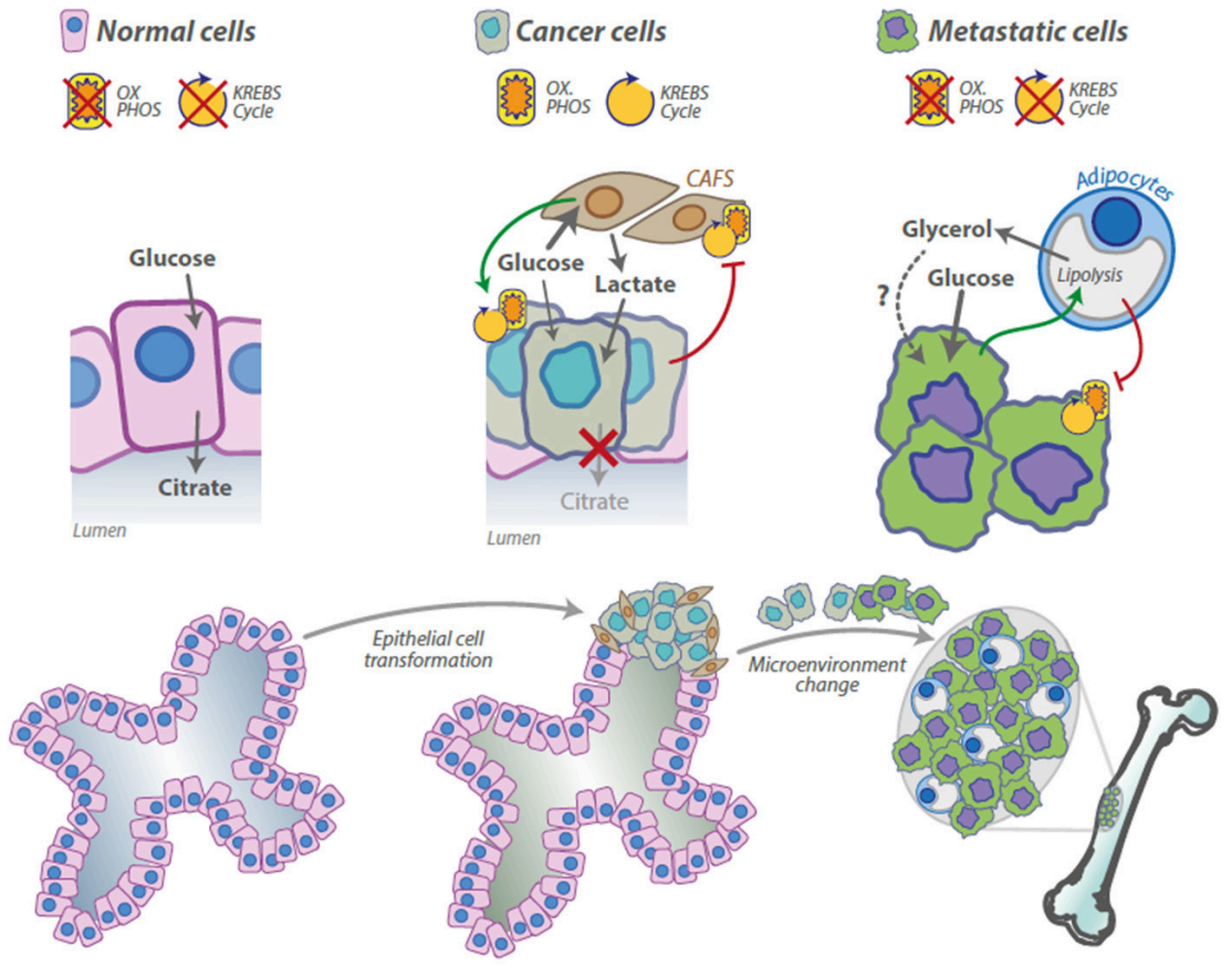
**Metabolic alteration in the tumor microenvironment during prostate cancer progression**. Normal prostate epithelial cells have a peculiar metabolic profile, the Krebs cycle and the OXPHOS are inhibited and the citrate is released as component of the seminal liquid. Prostate cancer cells corrupt cancer associated fibroblasts (CAFS) to activate the Warburg effect and to secrete lactate that is used by cancer cells for anabolic and catabolic processes, CAFS, in turn, induce OXPHOS activation in cancer cells. Prostate cancer cells preferentially metastasize in the bone. Cancer cells stimulate lypolisys in the adipocytes that secrete glycerol; adipocytes, in turn, stimulate the Warburg effect in cancer cells. Glycerol uptake and consumption by cancer cells has been described elsewhere, but it has not been demonstrated in this model.

One of the main functions of the prostate gland is to produce and secrete large amounts of citrate, which is released from epithelial cells into the lumen (Figure 1). Citrate production is obtained through the activity of a specific type of glandular epithelial cells and is not observable in any other kind of cells. The metabolism of prostate epithelial cells is modified for this special function. In the mitochondria of most mammalian cells, the pyruvate produced by glycolysis is decarboxylated to generate Acetyl-CoA, which reacts with oxalacetic acid to produce citrate that is then oxidized through the Krebs cycle. On the other hand, in normal prostate epithelium, mitochondrial citrate oxidation is impaired, mainly by inhibition of mitochondrial aconitase (m-aconitase), the enzyme responsible for the first step of transformation of citrate in the Krebs cycle (see also below): in these cells, citrate is thus the final product of the glucose metabolism and not an intermediate (Costello and Franklin, 2006). Differently from other cells in which, at the end of the Krebs cycle, oxalacetic acid is regenerated, in prostate cells this is not possible and oxalacetic acid is produced from aspartate that is imported from the plasma through the specific receptor excitatory amino acid carrier 1 (EAAC1) (Franklin et al., 2006). It has been clearly demonstrated that, in prostate cells, Krebs cycle is essentially inhibited so these cells are energetically inefficient. Prostate cells therefore show a very low level of oxidative phosphorylation (OXPHOS), counterbalanced by an increased glycolitic rate to survive and sustain the citrate production.

As a substrate for Krebs cycle, citrate represents an attractive source of energy for a transformed prostate cell, which has an increased energy demand. Franklin and Costello proposed “the bioenergetic theory of prostate malignancy”: the observation of very low levels of citrate in prostate cancer led to the hypothesis that malignant cells, in order to resume an efficient energy-generating system, become able to oxidize citrate in order to produce ATP, thus completing the Krebs cycle and transforming a healthy metabolically-inefficient prostate cell into an energy efficient malignant cell (Figure 1). These authors also concluded that this alteration is an early event in the malignant progression, which even precedes the histological identification of the malignant cells (Cooper and Farid, 1964; Costello et al., 1997; Costello and Franklin, 2000).

As previously mentioned, the inhibition of the Krebs cycle in normal prostate cells, resulting in the accumulation and subsequent secretion of citrate, is mainly obtained through the inhibition of m-aconitase, due to the accumulation of zinc in these prostate cells (Costello and Franklin, 2006). Citrate concentration in normal prostate cells is around 12-fold higher with respect to blood plasma levels, reaching the extraordinary concentration of about 13,000 nanomoles per gram of wet tissue. Zinc concentration is possibly even higher, reaching the concentration of about 4,000 nanomoles per gram that is around 200-fold higher with respect to blood plasma concentration. Zinc accumulation in the mitochondria truncates the Krebs cycle by inhibiting m-aconitase and determining the peculiar metabolism of normal prostate cells (Costello and Franklin, 2006). It has been observed that zinc levels are dramatically reduced in prostate cancer cells (Tsui et al., 2006), inducing the reactivation of m-aconitase and of the Krebs cycle. These data are supported by the observation that also citrate concentration is strikingly reduced in prostate cancer cells. If normal prostate cells sacrifice 60% of the energy producing 14 ATP molecules per glucose molecule, prostate cancer cells, which completely oxidize citrate, produce additional 24 ATP molecules becoming energetically highly efficient.

Other than inhibiting m-aconitase, zinc produces other important effects. Zinc is responsible for apoptotic induction in prostate cancer cells, by inducing the release of cytocrome c from mitochondria, activating the caspases cascade and inhibiting the antiapoptotic protein NF-κB (Feng et al., 2002; Uzzo et al., 2002; Huang et al., 2006). Invasive and proangiogenic capabilities of metastatic prostate cancer cells are also inhibited by zinc subministration (Uzzo et al., 2006). Considering the anti-tumor effects of zinc administration in the cellular models a very recent review suggested to use zinc for the treatment of prostate and other type of cancer that exhibit zinc reduction (Costello and Franklin, 2017). Zinc uptake from the interstitial fluid in normal prostate cells is probably due to the activity of the zinc transporters hZIP1 and hZIP3 (Franklin et al., 2003). Interestingly the hZIP1 protein is expressed at a lower level in the prostate epithelium of afro-american men compared to the caucasian population, which could partially explain the higher susceptibility of afro-american population to prostate cancer (Rishi et al., 2003). The study demonstrated that hZIP1 downregulation in prostate malignant tissue is associated with reduced zinc concentration in the same area. Recent studies clarified the mechanism of zinc transporters downregulation in prostate cancer. Makhov et al. demonstrated that AP-2a, a transcription factor responsible together with AP-1 for hZIP1 and hZIP3 transcription is epigenetically silenced through promoter hypermethylation in DU145 and LNCaP cell lines (Makhov et al., 2011). This suggests that the efficacy of the drugs that inhibit promoter hypermethylation, like 5-azacytidine, observed in prostate cancer cells, can be due to the reactivation of genes involved in cancer cell metabolism like hZIP1 and hZIP3.

## Lactate shuttling and the tumor microenvironment

For each step in cancer progression, cells need to readjust bioenergetics and metabolism to sustain the new status. If citrate oxidation transforms highly inefficient prostate cells in more efficient transformed cells, we should consider that, being citrate oxidation a central metabolic process in normal non-prostatic cells, this adjustment is necessary but not sufficient for a cell to become really tumoral. Transformed prostate cells need an extra input in terms of energy or availability of molecular backbones for the anabolic processes. A critical step forward in malignant progression might be controlled by the interaction with and the support of other cells in tumor microenvironment, which may provide metabolic substrates (as lactate, see below) to be channeled by cancer cells into energy-producing and/or anabolic pathways.

For many years, studies on cancer metabolism have been focused specifically on cancer cells using homotypical populations. Otto Warburg, the pioneer of these studies, suggested to use homogeneous cell populations, like those obtained from ascites fluid, to obtain reliable results and probably he influenced for decades the approach to this kind of experiments. More recently, several studies demonstrated the importance of the compartmentalization and heterogeneity in tumor microenvironment, also relatively to metabolism. In this complex scenario, in the same tumoral mass, some cancer cells seems to be highly glycolitic, others have higher mitochondrial respiration rates (Sonveaux et al., 2008). Moreover other cell types, like cancer-associated fibroblasts (CAFS), can directly participate to the complex metabolic mechanism particularly in prostate cancer (Figure 1). Fibroblasts are the second most abundant cell population in the tumor mass. It has been proposed that these cells would be an important source of lactate to be imported into cancer cells to produce pyruvate or to fuel anabolic processes. Lactate is normally considered a waste product, but surprisingly, in 2008, Sonveaux et al. demonstrated in several cancer models with a particularly active OXPHOS that this molecule is preferred to glucose and that lactate uptake and consumption influence key functions in cancer cells like redox homeostasis and angiogenesis (Sonveaux et al., 2008; Dhup et al., 2012).

Transformed prostatic cells use IL-6 secretion to corrupt fibroblasts (Doldi et al., 2015). Tumor epithelium may also promote the downregulation of the tumor-suppressor p62 in stromal fibroblasts, leading to a decrease in mTORC1 activity and c-Myc expression, in turn producing impairment in the mechanism of metabolic detoxification and the subsequent release of ROS and IL-6. An autocrine loop promotes TGF-β and the induction of CAF phenotype, which further increases epithelial invasion and tumorigenesis (Valencia et al., 2014). “Activated” fibroblasts would turn on the Warburg effect increasing glucose consumption and generating large amounts of lactate that is secreted in the microenvironment as a “waste product.” The effect of CAFS on tumor cells involves Pyruvate kinase M2 (PKM2) activation. Exposure of prostate cancer cells to CAFS induces phosphorylation and oxidation of PKM2; after modification, the protein loses its metabolic function and acquire the ability to migrate into the nucleus, to interact with the hypoxia inducible factor 1α (HIF1α) and the protein DEC1, to inhibit transcription of a specific microRNA (mir-205) driving the metabolic conversion to OXPHOS of prostate cancer cells (Fiaschi et al., 2012; Giannoni et al., 2015). A similar mechanism has been described also in immune cells. In macrophages, after Toll like receptor 4 (TLR4) stimulation, HIF1α and the “inactive” form of PKM2 interact to induce a metabolic switch, yielding a completely different effect than that observed in prostate cancer cells, as macrophages acquire a Warburg-like phenotype necessary for their complete activation (Palsson-McDermott et al., 2015). These data indicate that, although the basic mechanism involved in the metabolic switch controlling Warburg/OXPHOS is common to different cell types and scenarios, molecular adaptors selectively expressed in each cell type in the specific context determine the final output.

## Monocarboxylate transporters in prostate cancer

Although with some discrepancy, it is commonly accepted that an important role in CAFS interaction with prostate cancer cells is fulfilled by monocarboxylate transporters (MCTs). MCT family is composed by 14 members, but only for 4 transporters (MCT1-4) a role in the transport of monocarboxylate molecules has been demonstrated. MCTs translocate across the plasma membrane a monocarboxylate molecule together with a proton with no direct energy input. As consequence, MCT activity is dependent on substrates concentration and proton gradient (Juel, 1997). Whereas MCT3 is specifically expressed in the retina and in the choroid plexus, MCT1, MCT2 and MCT4 have been demonstrated to transport important monocarboxylates like lactate, pyruvate, ketone bodies (acetoacetate, β-idroxybutyrate) in several tissues including prostate cancer (Halestrap and Price, 1999; Pértega-Gomes and Baltazar, 2014). During prostate cancer progression, the expression profile of MCT isoforms changes (Pértega-Gomes and Baltazar, 2014). MCT1 is expressed in normal and malignant prostate, MCT2 increases from normal gland to PIN and *in situ* carcinoma. On the other hand, MCT4 is only expressed in malignancy. While MCT1 and 2 transport a wider range of substrates, MCT4 role is specifically associated with the export of lactate in cells with high glycolytic rate. Recently Pértega-Gomes et al. (2014) performed an immunohistochemical analysis on 480 samples from prostate cancer patients studying the expression of MCTs and other metabolic proteins. They observed important differences between CAFS and cancer cells. MCT4 and carbonic anhydrase IX (CAIX) were preferentially expressed on CAFS, by contrast MCT1 was expressed specifically on tumor cells. These data suggest that MCT1 could be responsible for lactate uptake in cancer cells from the acidic microenvironment; the import of lactate helps to regenerate NADH and accelerate glycolysis. Unfortunately, since the authors could not demonstrate the presence of glucose transporters on CAFS, thus the final demonstration of this mechanism is still missing.

## Warburg effect in prostate cancer metastasis

Metastatization, the process by which cancer cells leave the original tumor site and migrate to other parts of the body, is probably the most complex. Over and above than acquiring the capability to escape from the primary tumor detaching from the rest of the tissue, digesting the matrix and invading the circulation, the cells spend time and “efforts” to learn how to survive in a novel, possibly hostile, microenvironment. The dissemination in a new context needs new and dramatic adaptations requiring several new metabolic competencies.

Differently to the primary tumor, the metabolic mechanisms that sustain the growth of prostate cancer metastasis have not been clearly elucidated, the androgen signaling seems to be involved in some extent particularly in androgen sensitive cells LNCaP and VCaP inducing both glycolysis and OXPHOS, but the molecular mechanisms and the cellular context have been only partially elucidated (Tennakoon et al., 2014). In contrast to other cancer cell types that develop a higher level of glucose consumption early in the transformation process, prostate cancer cells switch to the Warburg effect only in the metastatic stage, excluding the possibility to use advanced diagnostic procedures like standard FDG-PET scan for the detection of tumor in the early stages (Schöder and Larson, 2004; Testa et al., 2016).

The prostate, together with lung and breast are the most frequent sites of primary tumors, but cancer cells from these sites share another important characteristic, as they can disseminate in the bone and bone metastasis represents an indication of poor prognosis (Mundy, 1997). The 1-year mortality is dramatically higher in prostate cancer patients with bone metastasis with respect to those with no skeletal involvement (Nørgaard et al., 2010). The new microenvironment means also new interactions with new cell types that need to be corrupted in order to sustain tumor growth.

The adipocytes are the most abundant cells in the bone marrow (Hardaway et al., 2014): the number of these cells grows together with the risk of metastatic prostate cancer with age, obesity and metabolic disorders (Justesen et al., 2001; Strotmeyer and Cauley, 2007). Moreover, the adipocytes are highly metabolically active cells, able to promote tumor growth and increased aggressiveness of cancer cells that grow as bone metastasis (Justesen et al., 2001; Herroon et al., 2013; Templeton et al., 2015). Very recent studies reported that the adipocytes could be responsible for the switch to the Warburg effect of prostate cancer metastatic cells, being able to modulate metabolism as shown for CAFS in the primary tumor. Prostate cancer cells co-cultured with adipocytes switch to a high glycolytic rate, increasing HIF1α production in an oxygen independent manner. HIF1α accumulation is responsible for the transcription of the Warburg related genes, resulting in lactate accumulation and OXPHOS inhibition. Using the stimulation with conditioned media (CM), the authors clarified that the physical interaction between cancer cells and adipocytes is not necessary; however, they failed in the identification of the soluble factor(s) responsible for the switch. Interestingly, prostate cancer cells (Pca) exposed to the adipocyte CM alone do not switch to the Warburg metabolism, while, using the conditioned media from the Pca/adipocyte co-culture, a dramatic metabolic reprogramming occurs in Pca cells. On the other hand, adipocytes treated with Pca CM increase the levels of the adipose triglyceride lipase (ATGL) the key enzyme in the lipolytic process and secrete large amount of free glycerol, suggesting a Pca-induced lipolytic phenotype (Diedrich et al., 2016). Although it has not been directly demonstrated in this model, it is known that glycerol can enter in the glycolytic process and feed cancer cells (Vaughan, 1962; Langin, 2006; Maeda et al., 2008). Overall these data validate the idea that Pca cells corrupt the other cells in the microenvironment to generate metabolic intermediates that are in turn used by cancer cells. Thus, the microenvironment metabolically sustains the growth of the tumor, creating a specific niche in which cancer cells can survive and proliferate.

## Prostate cancer and inflammation

**Figure 2:**
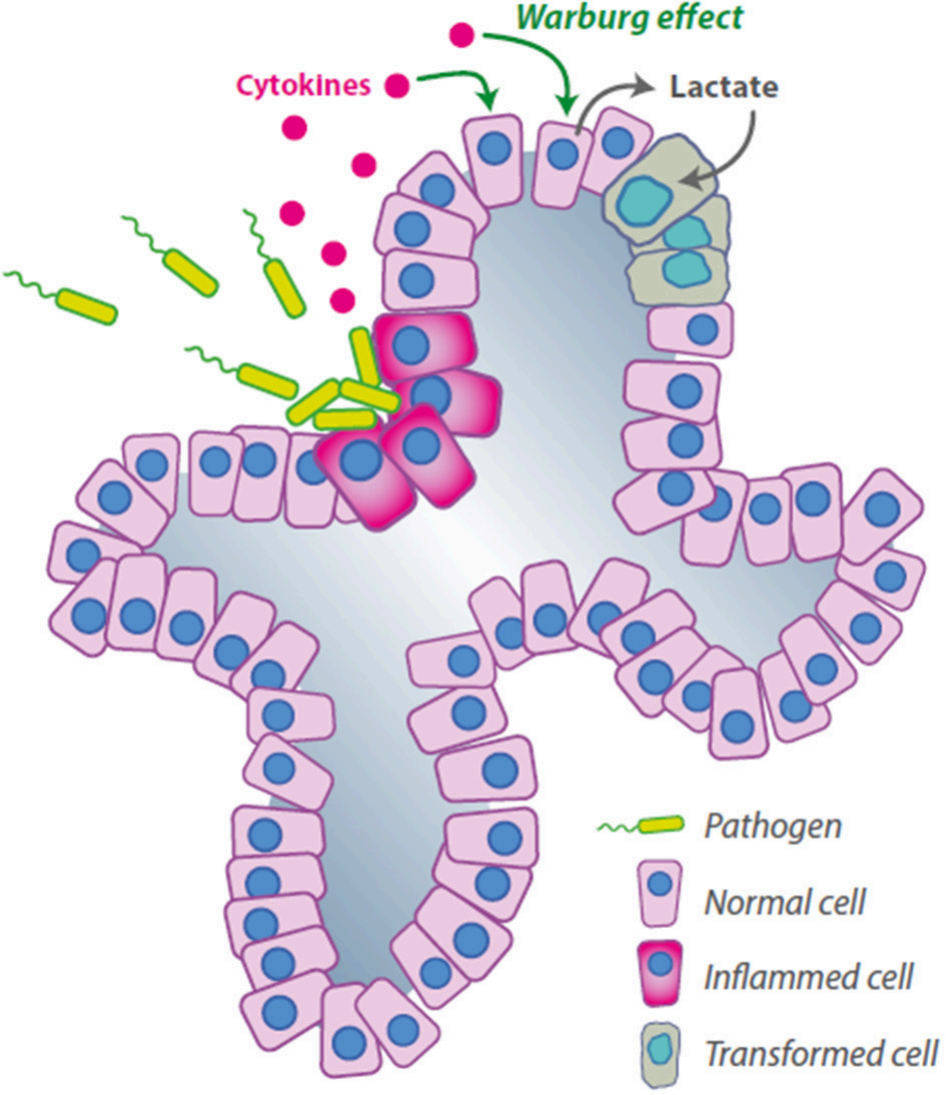
**Hypothetical mechanism of inflammation-induced cancer feeding**. The cytokines are released by infected cells and induce the Warburg effect in a different subpopulation, the lactate secreted would be used by transformed cells for anabolic and catabolic processes as described by Sonveaux et al. (2008).

About 20% of all human cancers are caused by chronic infection or chronic inflammatory states (De Marzo et al., 2007). Some of the roles of both innate and adaptive immunity in cancer progression have been clarified (Kim et al., 2009) but the complete mechanism is far from being elucidated. Although several criticisms have been raised on the detection protocols, a large number of clinical studies underline a positive correlation between recurrent prostatitis and prostate cancer (De Marzo et al., 2007). Unfortunately, the possible microorganism(s) or inflammation-generating factor(s) that should produce the chronic inflammatory status still remain elusive (Sfanos and De Marzo, 2012). A recent paper proposed a link between the immune system and the metabolic reprogramming of prostate cancer cells. Vaughan et al. (2013) describe the ability of the inflammatory factor Tumor Necrosis Factor α (TNFα) to induce a HIF1α-dependent metabolic switch in prostate cells, inducing aerobic glycolysis and blocking the OXPHOS mechanism. The authors in this study used the canonical prostate cancer cells lines LNCaP and PC3, but also tested RWPE cells that are commonly considered normal prostatic epithelium. As described before, metastatic cancer cells seem to acquire characteristics that predispose to the Warburg effect and in some manner this correlates with their ability to activate the aerobic glycolysis after TNF stimulation; surprisingly however, also RWPE show a Warburg phenotype after TNF stimulation (Vaughan et al., 2013). These data, if confirmed, would suggest a completely different track leading to metabolic reprogramming for those prostate cancer cells arising as a consequence of the chronic inflammatory status: in the inflammation-driven tumorigenesis, the Warburg phenotype would indeed be induced at the beginning of the transformation process (Figure 2). These would permit the identification of a new subset of patients that should be treated differently from the others maybe with anti-inflammatory therapies associated with the inhibition of glycolysis.

## Concluding remarks and open questions

The number of prostate cancer cases around the world is increasing. The incidence of this tumor has been associated with aging, environmental factors and changes in lifestyle. While it is becoming clear that a profound metabolic reprogramming occurs in prostate cancer cells, the majority of the molecular mechanisms involved in the process remain to be elucidated and are likely to be complex, encompassing changes within the prostate epithelial cells and crosstalk with other cell types present in the microenvironment. Although some data are available on the reshape of energy and carbohydrate metabolism, other metabolic pathways remain underinvestigated, as for example those involving amino acids (glutamine, serine) and fatty acids, as their control by epigenetic mechanisms, the latter proven to be important in many cancer types (Wong et al., 2017). Future research in the field of prostate cancer metabolism will also have to deal with elucidation of the interplay between inflammation and oncogenesis. There are also evidences that the metabolic reprogramming might follow different routes if inflammation-driven. The possible soluble signals coming from the pathogens leading to transformation of prostate epithelial cells are yet unknown as is their influence on the metabolism of epithelial cells or on inflammatory pathways in immune cells present in tumor microenvironment, which may contribute to speed up the malignant transformation.

The knowledge of the metabolic pathways and their regulation in prostate cancer will help to identify possible targets that can be exploited to device novel therapeutic strategies against prostate cancer. These targets could include proteins as glucose transporters, PKM2, the monocarboxylate trasporter MCT4 or the ZIP1 zinc transporter, as well as novel players to be identified in the next future.

## Author contributions

FC, GG, MM, AM, AP, SR, and APao studied the literature and critically revised the manuscript. FC and APao wrote the manuscript. FC, GG, and APao ideated the figures, GG prepared the figures.

## Funding

Funds from Associazione Italiana Ricerca sul Cancro to FC (AIRC-IG2015 n. 16720), from Regione Lazio (prog. FILAS-RU-2014-1020), from Fondazione Italiana Ricerca sul Cancro to MM (Triennal FIRC Fellowship Rif. 14843), from Sapienza University of Rome to AP (C26A149EC4) are gratefully acknowledged.

### Conflict of interest statement

The authors declare that the research was conducted in the absence of any commercial or financial relationships that could be construed as a potential conflict of interest. The reviewer MDR and handling Editor declared their shared affiliation, and the handling Editor states that the process nevertheless met the standards of a fair and objective review.

## Abbreviations

Pca: Prostate cancer
PIN: prostate intraepithelial neoplasia
OXPHOS: oxidative phosphorylation
HIF1α: hypoxia inducible factor 1α
m-aconitase: mitochondrial aconitase
CAFS: cancer-associated fibroblasts
DEC1: deleted in esophageal cancer 1
PKM2: Pyruvate kinase M2
MCTs: monocarboxylate transporters
CAIX: carbonic anhydrase IX
FDG-PET: FluDeoxyGlucose-Positron Emission Tomography
TLR4: Toll like receptor 4
TNFα: Tumor Necrosis Factor-α
TN breast cancer: Triple Negative breast cancer.
